# Establishment of local diagnostic reference levels for quality control in intraoral radiography

**DOI:** 10.1007/s11282-016-0245-9

**Published:** 2016-05-03

**Authors:** Maki Izawa, Yasuo Harata, Noriyoshi Shiba, Nobuhide Koizumi, Tomonori Ozawa, Nobutoshi Takahashi, Yasuhiko Okumura

**Affiliations:** Division of Dental Radiology, Department of Diagnostic and Therapeutic Science, Meikai University School of Dentistry, 1-1 Keyakidai, Sakadoshi, Saitama 350-0283 Japan

**Keywords:** Patient entrance dose (PED), Diagnostic reference level (DRL), Intraoral radiography, Radiation dosage

## Abstract

**Objective:**

To investigate the dosage and imaging conditions for patients undergoing intraoral radiography at Meikai University Hospital and establish assurance and quality control data.

**Methods:**

Tube voltage, exposure time, and air kinetic energy released per unit mass (air kerma) of three intraoral radiography units were measured. To calculate the patient entrance dose (PED) for each radiograph using Insight film, we extracted data for 1063 patients from their exposure records. The PED was compared with the diagnostic reference level (DRL) from the European Commission and the UK.

**Results:**

The tube voltage of the three units was maintained at 60 ± 2 kV. Differences in exposure time were less than 1.7 % for all units. The air kerma rates were well maintained within a 4.2 % error. Based on the patient data, there were no significant differences in the mean exposure times for males and females for all anatomical sites. The mean PED ranged from 1.09 ± 0.31 mGy for the mandibular incisors to 2.42 ± 0.33 mGy for the maxillary molars. The mean PED at the mandibular molars using InSight film was 1.59 ± 0.20 mGy, being less than the recommended value based on the DRL for intraoral radiography in the UK.

**Conclusions:**

We concluded that radiographic conditions at the hospital have been properly maintained. This basic quality control data may assist other dental radiation facilities to reduce patient dosage.

## Introduction

Dental treatment often requires diagnostic imaging using X-rays, and it is important that dental practitioners follow a system for radiation protection. Patient exposure to radiation must be kept suitably low, appropriate equipment and facilities must be used, and a quality assurance (QA) program needs to be in place.

According to the International Commission on Radiological Protection (ICRP) [1], the final responsibility for radiation exposure lies with the physician and, therefore, dental practitioners should always be trained in the principles of radiological protection, including the basic principles of physics and biology. Awareness of the proper patient dose is essential. To optimize diagnostic imaging based on the ICRP recommendations, dentists should observe the principle of ALARA (as low as reasonably achievable) [2]. Optimizing intraoral radiography, an essential element in dental care [3], should be a primary concern of every dentist charged with the safe and effective operation of their X-ray equipment.

It is recommended by the ICRP that patient dosage should be measured on a regular basis and compared with the diagnostic reference level (DRL) as part of a facility’s QA program [1, 4]. For an inspection under normal conditions, the DRL is used to determine a high value for an abnormal patient dose. For cases in which more extreme dosages are needed, it is necessary to investigate whether patient protection is optimized or at least sufficient [5, 6, 7].

On 1 April 2007, amendments to Japan’s Medical Care Act were enacted, and portions of Japan’s QA program were revised. Among the revisions, guidelines were developed to ensure the safety of medical care in both clinics and hospitals, including the implementation of training for employees in clinics without hospital beds. Thus, all facilities are required to ensure the safe use and maintenance of medical equipment, including quality control (QC) of X-ray imaging apparatuses.

Meikai University Hospital uses an analog system with non-screen-type film for intraoral radiography. The current equipment was introduced in March 2001. At that time, the irradiation dose was set on the basis of the technical parameters (radiographic information), such as radiographic equipment type and projection technique. Adult male maxillary anterior teeth were used as the standard.

The purpose of this study was to optimize intraoral X-ray image diagnosis in the dental practice of Meikai University Hospital. Another aim was to obtain basic information as part of QC by comparing our radiography conditions and patient doses with the general DRL.

## Materials and methods

### Materials

Intraoral radiography in the Meikai University Hospital Radiology Department is undertaken using three Heliodent 60DS X-ray units (Sirona, Bensheim, Germany). The outputs of the units were measured using a ThinX RAD test device (Unfors, Billdal, Sweden), which displays the tube voltage, half-value layer, exposure time, air kinetic energy released per unit mass (kerma), and air kerma rate at the cone tip. To investigate the exposure factors for intraoral radiography using InSight X-ray film (Kodak/Carestream, Rochester, NY, USA), the exposure records of patients at Meikai University Hospital during 1–30 April 2011 were extracted. Based on the air kerma without backscatter at the cone tip, the patient entrance dose (PED) was computed for each exposure factor [8]. The Meikai University Department of Dentistry Ethics Committee approved this study (A1101).

### Output X-ray units

The department’s intraoral radiographic system using InSight film, Heliodent 60DS X-ray units, and DENT-X Type 810 automatic processors (AFP, New York, NY, USA) was introduced in March 2001. At that time, the exposure factors were determined by reference to the previous Ekta-Speed Plus system (Kodak/Carestream). However, studies of exposure factors were not fully carried out for the new system. In the present study, we aimed to establish local DRLs for the new system and determine the baseline for QC of the system to set the initial values for the exposure factors. The current-exposure times of F group films taken with a 20-cm standard cone based on the Heliodent 60DS manual are shown in Table 1 for projection site, male or female sex, and/or adult or child patients. The specification of the Heliodent 60DS units was as follows: high-frequency rectification of tube voltage of 60 kV, fixed tube current of 7 mA; 2-mm total filtration of aluminum equivalent (Al eq.), and timer setting from minimum 0.01 s to maximum 3.2 s.

**Table 1:** Specification of the Heliodent 60DS units and exposure times recommended by the manufacturer

Patients	Projection	Sex	Incisor (s)	Premolar (s)	Molar (s)
Adults	Maxillary	Male	0.32	0.40	0.50
		Female	0.25	0.32	0.40
	Mandibular	Male	0.16	0.20	0.32
		Female	0.12	0.16	0.25
	Occlusal	Male	0.64		
		Female	0.50		
Children	Maxillary		0.12	0.20	0.25
	Mandibular		0.06	0.10	0.12
	Occlusal		0.40		

The sensor of the ThinX RAD test device was set at the cone tip. The focus-cone tip distance was 20 cm. X-Omat X-ray film (Kodak, New York, NY, USA) was placed in the same position, and the exposure field was measured. We measured the tube voltage, aluminum half-value layer, exposure time (actual measurement), air kerma, and air kerma rate for each exposure. The measurements were performed three times, and mean values and standard deviations were computed for each unit.

### Intraoral radiography and PED

In the present study, the intraoral X-ray films, projection techniques (periapical bisecting, paralleling, bitewing, occlusal), and exposure factors (tube voltage, tube current, exposure time) for the exposure records of 1063 patients (474 males, 589 females) were surveyed by certified and authorized radiologists of the Japanese Society for Oral and Maxillofacial Radiology.

Using two DENT-X Type 810 automatic processors that were fully managed by two radiologists, the films were developed and fixed with DENTX RCDF solution (AFP). When a film met the diagnostic criteria, the PED was calculated as follows:

1 $${\text{PED }} = \, \alpha \left[ {{\text{mGy}}/{\text{mAs}}} \right] \, \times {\text{ mAs}}$$

where *α* is the air kerma without backscatter at the cone tip per mAs.

The mean (±SD) and 75th percentile of the PED distributions were compared with the DRLs of intraoral radiography from the European Commission (EC) [8] and the UK [2].

### Sex differences in PEDs

We compared the mean PEDs of male and female patients using the bisecting technique for each anatomical site using the Tukey–Kramer test after checking the normality of each series of data and uniformity of variance among all series. We determined the significance level for statistical differences in the PEDs to be 5 %.

## Results

### Intraoral X-ray units

**Fig. 1:**
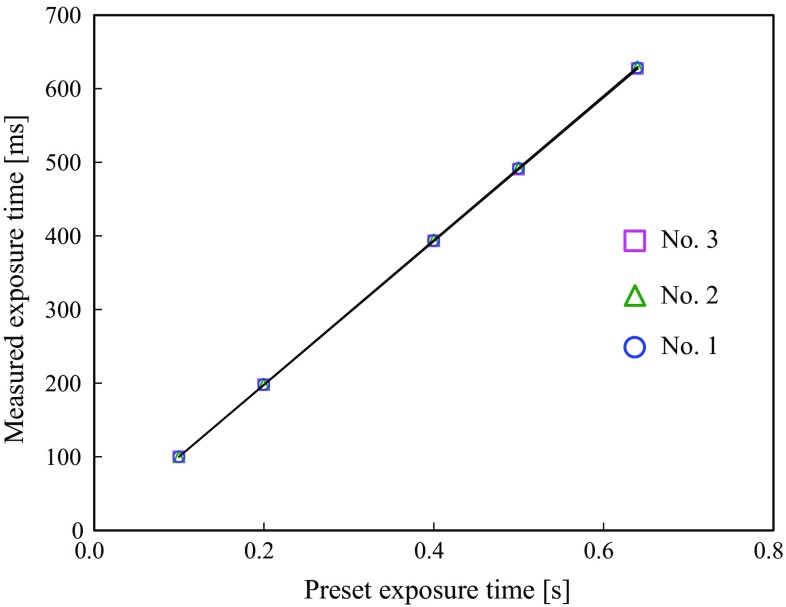
Exposure time measured as a function of preset exposure time for three intraoral X-ray units in Meikai University Hospital Radiology Department

For all timer settings of the X-ray units, the tube voltage was 60 ± 2 kV, and the half-value layer was 2.0-mm aluminum with a 95 % confidence interval (CI). The coefficient of variation was 2 % for exposure time of *Y* (ms) at timer setting of *X* (s) with relationships of *Y* = 982.99*X*, *Y* = 981.26*X*, and *Y* = 984.82*X* for units 1, 2, and 3, respectively. The differences in the exposure times of the units were less than 1.7 %. The resultant air kerma rates were constant (within 4.2 %) with a 95 % CI (Fig. 1). The diameters of the exposure fields at the cone tip were the same for all units (57 mm).

### Timer settings and PEDs

**Fig. 2:**
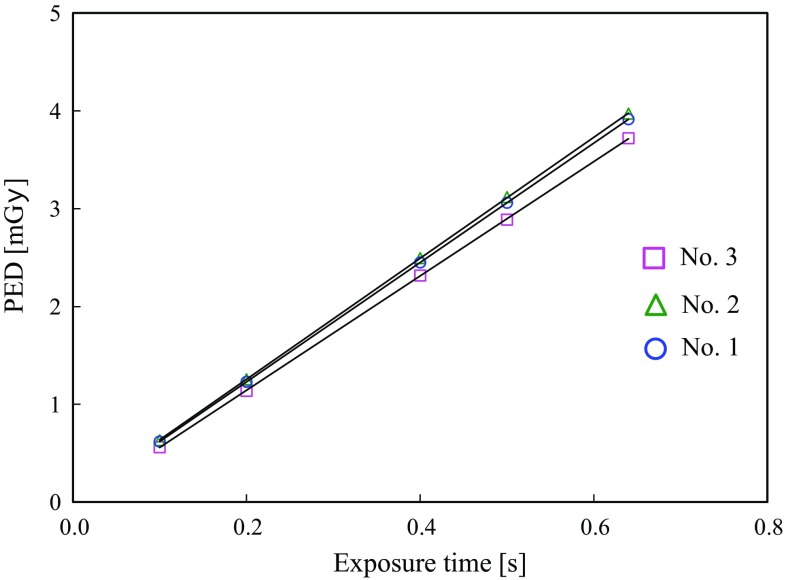
Air kerma at the cone tip end in free air as a function of preset exposure time

The air kerma at the cone tip was well correlated with the timer settings for the units (Fig. 2). From linear regressions, the *α* values in Eq. (1) were 0.874, 0.889, and 0.827 Gy/mAs (mean: 0.863 mGy/mAs). We used the mean value of α to estimate each PED, because we could not specify the unit used for each patient from the exposure records.

### PEDs in intraoral radiography

**Fig. 3:**
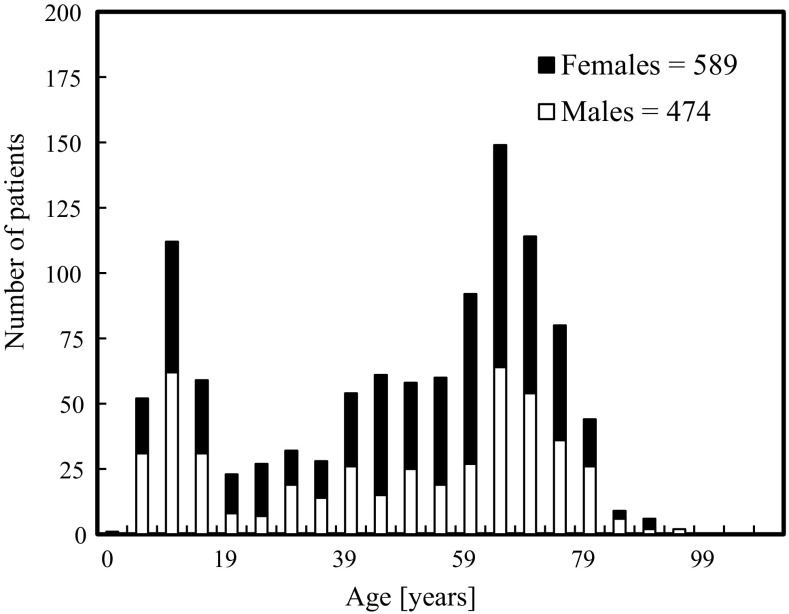
Age distribution of patients undergoing intraoral radiography in Meikai University Hospital Radiology Department

**Fig. 4:**
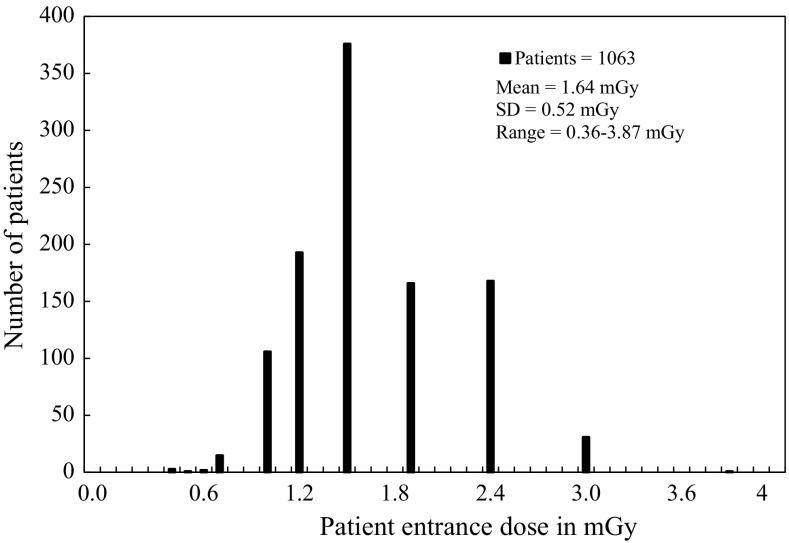
Patient entrance dose distribution for all patients undergoing intraoral radiography in Meikai University Hospital Radiology Department

Figure 3 shows the sex and age distributions of the patients in the present study. There were more female patients than male patients (sex ratio 2:3), and 75 % of the patients were adults above the age of 18 years. The intraoral radiography techniques investigated were the periapical bisecting (94 %), bitewing (5 %), and occlusal (1 %) techniques. The mean exposure time for all patients was 0.27 s, with a minimum of 0.06 s and a maximum of 0.64 s. The mean (±SD) PED (*σ*_p_) was 1.64 ± 0.52 mGy. The PED values ranged from 0.36 to 3.87 mGy, and varied by a factor of up to 10 depending on the projection technique, anatomical site, and sex and age of the patient. The PED distribution is shown in Fig. 4.

### Exposure times and PEDs for the periapical bisecting technique

**Fig. 5:**
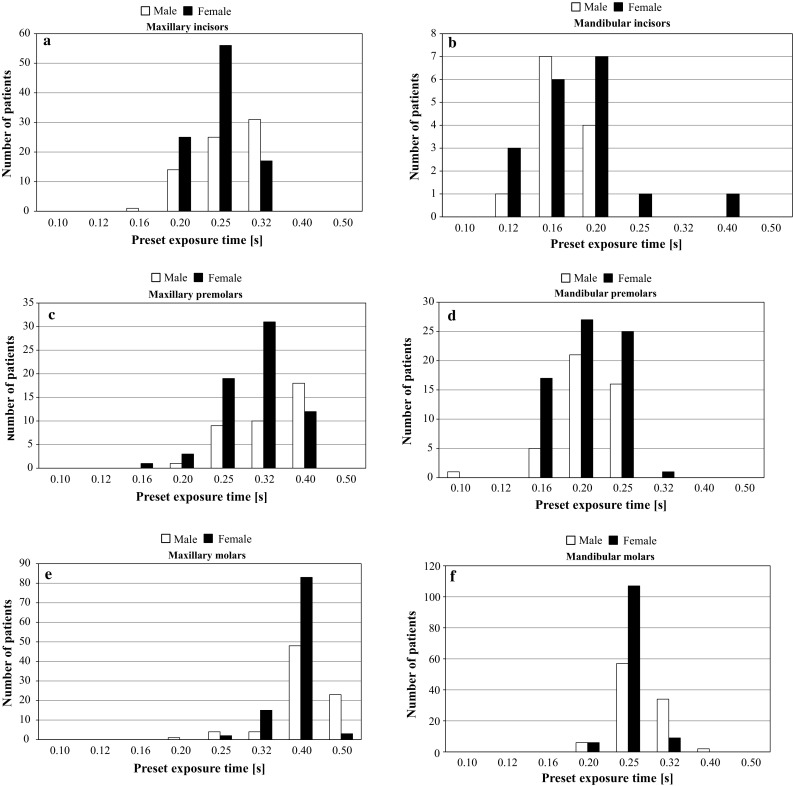
Exposure time distributions for patients undergoing periapical bisecting technique radiography in Meikai University Hospital Radiology Department. **a** Exposure time distribution for maxillary incisors. **b** Exposure time distribution for mandibular incisors. **c** Exposure time distribution for maxillary premolars. **d** Exposure time distribution for mandibular premolars. **e** Exposure time distribution for maxillary molars. **f** Exposure time distribution for mandibular molars

We performed statistical analyses of the exposure times and PEDs for the most frequently used radiographic technique (i.e., the periapical bisecting technique). Table 2 shows the mean PEDs of the male and female adult patients separately for each anatomical site (6 sites × 2 sexes = 12 series). Figure 5a–f shows the exposure time distributions for the male and female adult patients in detail. The distributions showed normality for each series, and uniform variance, checked using the Bartlett test, for all series. The Tukey–Kramer test showed that the mean exposure times for male and female patients did not differ significantly for all anatomical sites. To more easily manage the exposure times and patient doses, the PED values were averaged, ignoring the sex differences. The mean (±SD) PEDs for both male and female patients were 1.56 ± 0.27 mGy for maxillary incisors, 1.92 ± 0.38 mGy for maxillary premolars, 2.42 ± 0.33 mGy for maxillary molars, 1.09 ± 0.31 mGy for mandibular incisors, 1.27 ± 0.22 mGy for mandibular premolars, and 1.59 ± 0.20 mGy for mandibular molars. The 75th percentile values of the exposure times and PEDs are shown in Table 2 for each anatomical site.

**Table 2:** Exposure times and patient entrance doses (PEDs) for the periapical bisecting technique

Site	Sex	Patient (*n*)	Exposure time (s)^a^		Current-exposure time product (mAs)	PED (mGy)^b^
Maxillary incisors	Male	71	0.27 ± 0.05	0.26 ± 0.04 (0.32)	1.80	1.56 ± 0.27 (1.93)
	Female	98	0.25 ± 0.04			
Maxillary premolars	Male	38	0.34 ± 0.07	0.32 ± 0.06 (0.40)	2.23	1.92 ± 0.38 (2.42)
	Female	66	0.31 ± 0.06			
Maxillary molars	Male	80	0.41 ± 0.07	0.40 ± 0.06 (0.40)	2.80	2.42 ± 0.33 (2.42)
	Female	103	0.39 ± 0.04			
Mandibular incisors	Male	12	0.17 ± 0.02	0.18 ± 0.05 (0.20)	1.26	1.09 ± 0.31 (1.21)
	Female	18	0.19 ± 0.06			
Mandibular premolars	Male	43	0.21 ± 0.04	0.21 ± 0.04 (0.25)	1.47	1.27 ± 0.22 (1.51)
	Female	70	0.21 ± 0.04			
Mandibular molars	Male	99	0.27 ± 0.04	0.26 ± 0.03 (0.25)	1.84	1.59 ± 0.20 (1.51)
	Female	122	0.25 ± 0.02			

No significant difference between male and female.

^a^Mean exposure time ± standard deviation with 75th percentile in parentheses.

^b^Mean patient entrance dose ± standard deviation with 75th percentile in parentheses. The 75th percentile is the initial value of the local diagnostic reference level

As we explain later in the “Discussion”, well-trained radiologists or radiographers usually select the exposure time depending on the size of the patient. Because female adult patients are generally smaller than males, operators tended to select a shorter exposure time for female adults than for male adults.

## Discussion

The DRL is based on a standard physique or phantom that is widely used in standard radiographic inspections, and is not tailored to the individual [1]. Sakaino and colleagues [9], who investigated patient doses in intraoral radiography in a general dentistry clinic, suggested that it was not effective to examine a protocol and radiographic conditions more than once to establish a DRL. The DRL recommended by the United Kingdom [10] for imaging the mandibular molars was also not distinguished by sex. A comparison of the DRL recommended by the United Kingdom with a DRL based on the ICRP Supporting Guidance [5] should also be taken into consideration in the present study. Therefore, we examined the periapical bisecting technique in adult patients whose physical sizes were relatively constant and who undergo radiography frequently.

In this investigation, as a result of measuring the air kerma of the equipment with an exposure field 57 mm in diameter at a focus-cone tip distance of 20 cm, the sex-unrelated mean PED value was 1.59 ± 0.20 mGy in the mandibular molars. This value is <2.1 mGy, which is the 75th percentile of the PED under the same conditions used in the United Kingdom [10]. Compared with the PED value at the time of an investigation conducted in 2007 on the periapical bisecting technique in an adult patient using the same radiographic devices combined with Ekta-Speed film [11], the results of this investigation showed a 20 % reduction in dose. It has been proven and officially verified that Kodak’s more sensitive InSight film can create a radiographic image with a 20 % smaller dose of radiation compared with Ekta-Speed Plus film [12]. Thus, use of high-sensitivity film has brought about a reduction in patient dose in the Meikai University Hospital Radiology Department.

Measuring the patient dose and setting the baseline, remedial level, and suspension level for that patient, in combination with a QA program that monitors patient dose, would go a long way toward preventing unnecessary and inefficient work. Moreover, it would be useful for ensuring radiological safety by creating awareness of potential high-risk situations [13–16].

If the 75th percentile value of the PED distribution is set as the initial baseline value, it would have a mean value ± 0.6745*σ* (*σ*: standard deviation) for a normal distribution (the mean value ± *σ* are 84th percentiles). In fact, the 75th percentile value was 11–26 % more than the mean value in other areas and almost the same as the mean value in the maxillary and mandibular molars.

Therefore, we took the mean value of the PED (1.59 ± 0.20 mGy) as the baseline for QC in this radiographic system. The baseline ±20 % was set as the remedial level and the baseline ±40 % was set as the suspension level [8, 9, 13]. This was equivalent to a range of *σ* and 2*σ* in each part, being about ±20 and ±40 %, respectively. For the mandibular molars, with a mean PED of 1.59 mGy as the baseline, the remedial level of 20 % and suspension level of 40 % were 1.98 and 2.23 mGy, respectively. Although the value of the remedial level was <2.1 mGy, the value of the suspension level was >2.1 mGy. When the mean PED becomes greater than the remedial level, the radiographic conditions causing the change in the mean PED should be investigated, although use may continue. When the mean PED becomes greater than the suspension level, unless continuing radiographic imaging is justified, radiographic imaging should be stopped until conditions improve.

Thus, setting up a local DRL for each institution with a radiographic device on site is the first step toward reducing patient radiation exposure. This approach will be indispensable for ensuring safe and reliable X-ray diagnostic imaging.

This investigation confirmed that the radiographic conditions in the Meikai University Hospital Radiology Department have been properly maintained, but the local DRL still needs to be improved periodically. This includes adopting remedial and suspension levels as initial values [5]. To optimize radiographic conditions in the future, in addition to providing unified radiography training and continuing to study patient doses, investigations focused on maintaining QA/QC in an analog system, including evaluations of image quality and development conditions, are necessary. A digital system for evaluating the dose reduction effect also needs to be introduced.

## Conclusions

We investigated the exposure conditions for intraoral radiography in the Meikai University Hospital Radiology Department. The results are summarized as follows.

1. The tube voltage of the three units was 60 ± 2 kV, and the preset time of the timer was measured with an error of 1.7 % within a range of 0.1–0.64 for all units. The air kerma at the cone tip was 0.863 mGy/mAs at a nominal tube current of 7 mA (error <4.2 %).
2. Of the intraoral radiography subjects in the investigation, more than 70 % were adult patients over the age of 18 years. Their mean PEDs were 1.56 ± 0.27, 1.09 ± 0.31, 1.92 ± 0.38, 1.27 ± 0.22, 2.42 ± 0.33, and 1.59 ± 0.20 mGy (about 20 % SD) for the maxillary incisors, maxillary premolars, maxillary molars, mandibular incisors, mandibular premolars, and mandibular molars, respectively.
3. In the Meikai University Hospital Radiology Department, even considering the SD, the mean PED at the mandibular molars using InSight film was 1.59 ± 0.20 mGy, which was less than the recommended value.

Therefore, we concluded that exposure conditions at the hospital were properly maintained. Our findings could help with establishing local DRLs at other institutions, which would be important for optimizing patient protection.
